# Localization and Androgen Regulation of Metastasis-Associated Protein 1 in Mouse Epididymis

**DOI:** 10.1371/journal.pone.0015439

**Published:** 2010-11-03

**Authors:** Li Ma, Wei Li, Hua-Ping Zhu, Zhen Li, Zhi-Jian Sun, Xin-Ping Liu, Jie Zhao, Jin-Shan Zhang, Yuan-Qiang Zhang

**Affiliations:** 1 Department of Human Anatomy and Histology and Embryology, the Fourth Military Medical University, Xi'an, People's Republic of China; 2 Department of Biochemistry and Molecular Biology, the Fourth Military Medical University, Xi'an, People's Republic of China; Indiana University School of Medicine, United States of America

## Abstract

**Background:**

Metastasis-associated protein 1 (*MTA1*), the founding member of the MTA family of genes, can modulate transcription by influencing the status of chromatin remodeling. Despite its strong correlation with the metastatic potential of cancer cells, MTA1 can also regulate crucial cellular pathways by modifying the acetylation status. We have previously reported the presence of *MTA1*/MTA1 in human and mouse testes, providing the evidence for its involvement in the regulation of testicular function during murine spermatogenesis. The objective of present study was to further assess the localization of MTA1 in mouse epididymis on both transcriptional and translational level, and then to explore whether MTA1 expression is regulated by androgens and postnatal epididymal development.

**Methodology/Principal Findings:**

Mice were deprived of circulating androgen by bilaterally castration and were then supplemented with exogenous testosterone propionate for one week. MTA1 was immunolocalized in the epithelium of the entire epididymis with the maximal expression in the nuclei of principal cells and of clear cells in proximal region. Its expression decreased gradually after castration, whereas testosterone treatment could restore the expression, indicating that the expression of this gene is dependent on androgen. During postnatal development, the protein expression in the epididymis began to appear from day 7 to day 14, increased dramatically from postnatal day 28, and peaked at adulthood onwards, coinciding with both the well differentiated status of epididymis and the mature levels of circulating androgens. This region- and cell-specific pattern was also conservative in normal human epididymis.

**Conclusions:**

Our data suggest that the expression of MTA1 protein could be regulated by androgen pathway and its expression level is closely associated with the postnatal development of the epididymis, giving rise to the possibility that this gene plays a potential role in sperm maturation and fertility.

## Introduction

Mammalian epididymis is a highly specialized male reproductive organ and it can be grossly divided into the initial segment, caput, corpus and cauda on the basis of histological and structural differences [1]. Regional differences along the epididymis are essential for the establishment of the microenvironment required for germ cell maturation [2]. It has also been confirmed that epididymis is an androgen-responsive organ and many functional genes within the different regions of the epididymis have been identified to be under the influence of androgens. Variations in the distribution of different genes along specific regions of epididymis affect the environment in which spermatozoa acquire fertilizing ability and motility through the absorption, secretion, synthesis, and metabolism of substances that contact sperm [3]–[5]. Therefore, the identification and analysis of the different patterns of gene expression and their relationship to the androgen pathway along the long convoluted tubule may provide valuable information about the role of these genes in spermatozoa maturation.

*MTA1*/MTA1, a constituent of the nucleosome-remodelling and –deacetylation (NuRD) complex, was originally isolated by differential cDNA hybridization from rat mammary adenocarcinoma [6]. Its expression has previously been found to be associated with progression of the tumor invasion and metastasis [7], [8]. However, intriguingly, evidence supports that additional, yet uncharacterized role of MTA1 in reproduction is likely to take place [9]. In previous work, we have systematically examined the expression of MTA1 in adult mouse tissues [10], especially in testis [11]–[12]. Positive signals were observed on variety of tissues/cells in multiple systems including nervous, cardiovascular, respiratory, digestive, immune, endocrine, urinary, reproductive and sensory organ systems at both mRNA and protein levels. In addition, MTA1 protein is gradually increased in the testis since 14 days postnatal and reaches the maximum in adults. Immunolocalization analysis demonstrates that MTA1 is predominantly present in the nuclei of primary spermatocytes and spermatogonia. The most intensive staining is localized in the nuclei of pachytene spermatocytes in mature testes, suggesting its possible role during meiosis. More recently, we also demonstrated that overexpression of MTA1 in vitro could remarkably elevate the capability of spermatogenic tumor cells against heat-induced apoptosis, with a marked impairment of p53 expression [13]–[14]. Collectively, these observations strongly indicate that MTA1 expression may be indispensable for reproductive function.

In continuation to the above basis, we aimed to immunohistochemically localize MTA1 to the various cell types within the segments of mouse and human epididymis, and to determine on the one hand whether MTA1 expression is region-specific and on the other hand whether this expression is regulated by androgens and postnatal epididymal development. In the second experimental setting, we also characterized the expression pattern of MTA1 in mouse epididymis after bilateral castration using western blot analysis, RT-PCR and immunohistochemistry assay. The data obtained here may gain further insight into the potential function of this histone modifier in male reproduction.

## Results

### Region- and Cell-Specific Expression of MTA1 in Adult Mouse Epididymis

**Figure 1:**
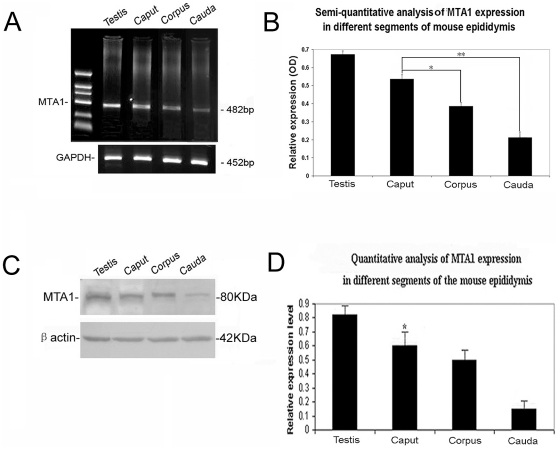
Expression level of MTA1 in different segments of adult mouse epididymis. (A) The products of a representative semi-quantitative RT-PCR were subjected to electrophoresis on a 1.5% agarose gel. *GAPDH* was used as an internal control for each PCR amplification. (B) Relative expression levels in RT-PCR were obtained in each sample by normalization of absolute optical densities (ODs) of the specific target to that of the *GAPDH* signal. (C) Western analysis of MTA1 protein in total tissue protein extracts from caput, corpus and cauda of the epididymis and testis. The blot was reprobed with a β-actin (42 kDa) monoclonal antibody to confirm equal loading of proteins in all lanes. (D) Semi-quantitative values are normalized to those of loading controls (β-actin) to express arbitrary units of relative expression. Comparison of the relative densities between groups in both assays was performed by ANOVA. (* p<0.05 and ** p<0.01).

MTA1 was expressed in all different segments of epididymis at both transcriptional and translational levels. The semi-quantitative RT-PCR was firstly performed to quantitate the expression level of *MTA1* mRNA in different segments of epididymis. For a better analysis, the number of cycles was tested to optimize amplification in the exponential phase of PCR as described elsewhere [10]. Analysis of intensity of PCR signals as function of the number of amplification cycles revealed a strong linear relationship between cycles 28 and 36, with a correlation coefficient r^2^ = 0.937. Moreover, a strong exponential (log-linear) relationship between signal intensity and cycle number was observed for less than 34 cycles. Thus, considering the diversity of experimental samples to be tested, 32 PCR cycles were selected for semi-quantitative analyses of *MTA1* mRNA levels. As shown in Fig. 1A, there was a clear decreasing expression tendency along the three segments, with the highest expression of *MTA1* in caput. Semi-quantitative analysis of the band density using Image J software revealed statistical significances between caput and corpus (*p = 0.0217 <0.05), and between caput and cauda (**p = 0.0034 <0.01), respectively (Fig. 1B). No bands were observed in the negative control (data not shown). As expected, these results were in good agreement with the western blot analysis (Fig. 1C, D).

**Figure 2:**
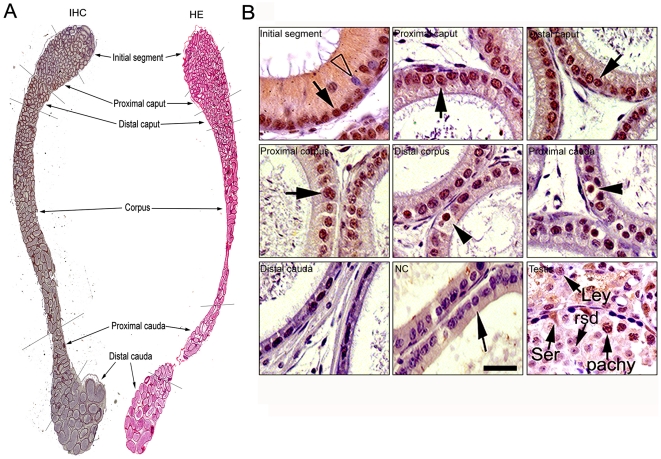
The localization of MTA1 protein in the adult mouse epididymis. (A) The immunohistochemical staining showed the region-specific expression pattern of MTA1 protein in the whole epididymis even at a lower magnification. Gradually decreased expression was observed from initial segments to the distal cauda. Original magnification ×4 (B) Higher view of IHC staining revealed a detailed expression pattern. MTA1 was mainly localized in the nuclei of principal cells (black arrows) and of clear cells (black arrow heads) with the maximal expression level along initial segment, caput and proximal corpus. The narrow cells (Empty arrow heads), the luminal contents and the intertubular space were all completely negative for MTA1 staining. Staining of testicular section was served as positive control. All sections were slightly counter-stained by hematoxylin. Abbreviations: Ser, Sertoli cell; Ley, Leydig cell; pachy, pachytene spermatocyte; rsd, round spermatid. Bar = 10 µm.

To further elucidate the expression pattern of MTA1 in epididymis, we next carried out an immunohistochemical analysis. Positive signals were almost detected in the epithelium of the entire adult mouse epididymis as demonstrated in a low magnification (Fig. 2A). Overall, the expression level of MTA1 protein was higher in the initial segment, caput and proximal corpus than in the other segments. Positive staining could be barely detected in caudal region, especially in distal cauda. In epithelial cells, staining was most concentrated in the nuclei of principla cells (*Black arrows*) and clear cells (*Black arrow heads*) while narrow cells (*Empty arrow heads*), the luminal contents and the intertubular space were all completely negative. The mature sperms inside the lumen of epididymis were not stained either, when compared to the clear staining in the nuclei of epithelial cells. Moreover, the level of MTA1 protein expression progressively reduced from the caput and corpus to the cauda epididymidis even at a high magnification (Fig. 2B). Replacement of the primary antibody with normal goat IgG abolished the immunostaining in the tissues, confirming the specificity of immunohistochemical detection. Testicular expression of MTA1 was served as positive control [12].

**Figure 3:**
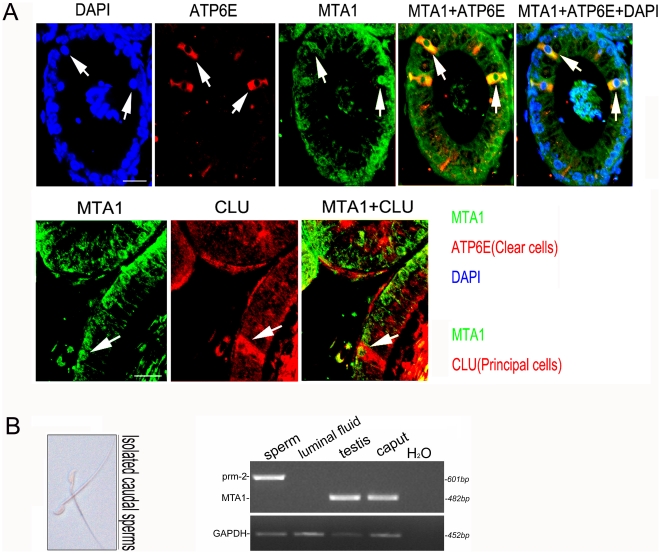
Cellular localization of mouse MTA1 in different cell types of the adult mouse epididymis. (A) The subcellular localization of MTA1 protein was determined by using cell-specific antibodies. The immunofluorenscence of MTA1 (FITC-labeled, green), ATP6E (Rhodamine labeled for clear cells, red), CLU (Rhodamine labeled for principal cells, red) and their colocalization in the distal caput were shown by confocal microscope. DAPI was used to stain nuclei of epithelium. Bar = 20 µm (B) RT-PCR analysis of *prm-2* (601 bp) and *MTA1* (482 bp) expression was conducted in isolated caudal sperm and luminal fluid, with testis and caput as positive controls. Left penal depicted representative isolated caudal sperms.

To further identify the different cell types that express MTA1, we employed the following antibodies to immunolocalize MTA1 protein in different cell types: anti-ATP6E IgY for clear cells, anti-F48 antibody for basal cells, and anti-CLU antibody for the principal cells [15]. The results of the confocal immunofluorescent staining of the same or sequential sections (Fig. 3A) showed that MTA1 was mainly accumulated in the nuclei of principal cells and clear cells but not in basal cells (data not shown). Very strangely, we also found a comparable positive staining of MTA1 inside the epididymal lumen. To convincingly address the question of whether this was unspecific signal, we isolated the caudal spermatozoa and luminal fluid and thereafter conducted RT-PCR analysis. As clearly demonstrated in Fig. 3B, a sharp band of 482 bp could only be detected in testis and caput cDNA samples with complete absence in both spermatozoa and luminal fluid, thus yielding a conclusion consistent with the previous IHC assay.

### Region-Specific Expression of MTA1 Is Conservative in Human Epididymis

**Figure 4:**
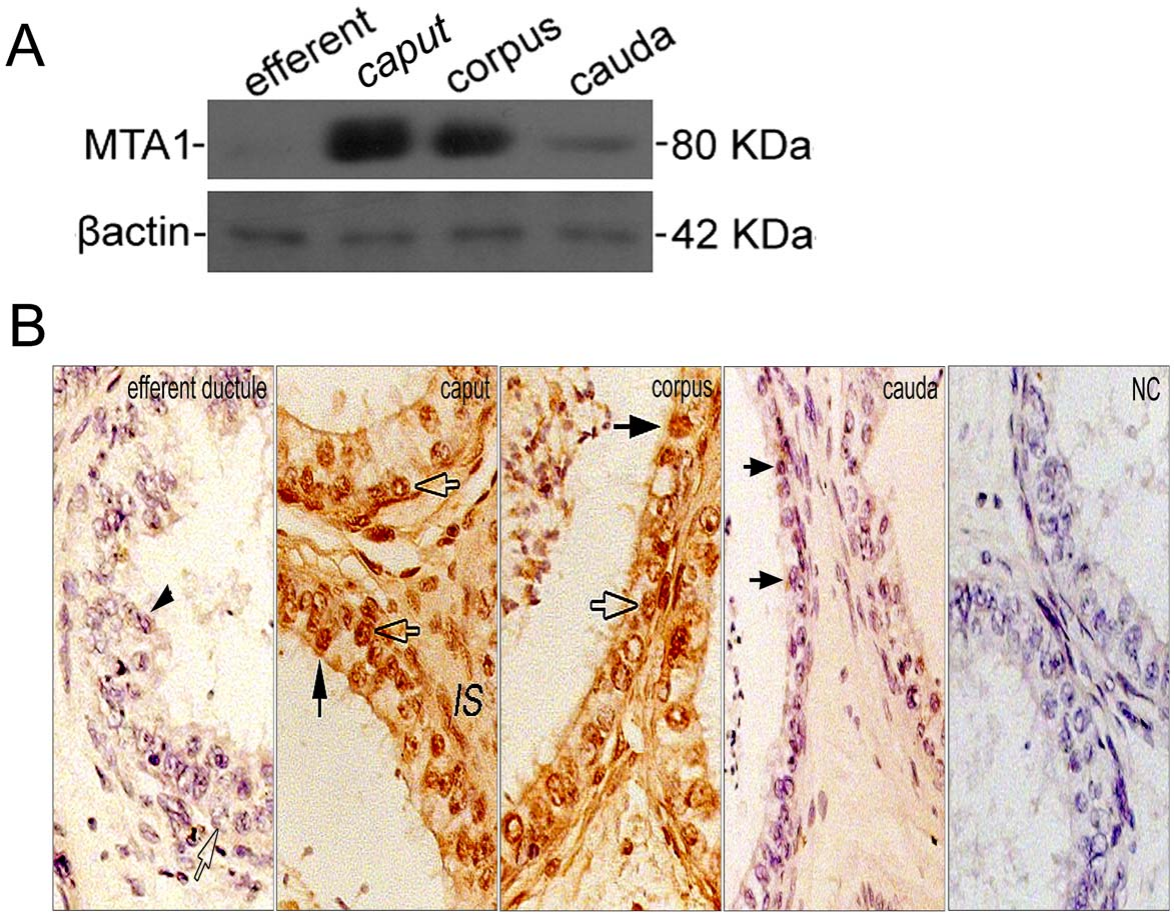
Characterization of MTA1 expression in normal human excurrent duct system. (A) Lysates from human efferent ductules, caput epididymis, corpus epididymis and cauda epididymis were separated over 10% SDS-PAGE and analyzed by immunoblotting with anti-MTA1 polyclonal antibody, respectively. For control purposes, loading of tissue extract for SDS-PAGE was corrected for levels of β-actin. (B) Comparative analysis of the expression of MTA1 in different segments of excurrent duct system by immunohistochemistry. The positive cells were identified as ciliated principal cells of epididymis (arrows) and basal cells (empty arrows). Columnar ciliated cells of efferent ductule (arrow heads) were barely stained. (*IS*, interstitial space) Negative control was performed using a nonimmune serum instead of primary antibody as demonstrated in NC. Bar  = 20 µm.

Usually, functionally less important molecules or parts of a molecule evolve (in terms of mutant substitutions) faster than more important ones [16]. To this point, we evaluated the MTA1 expression in human excurrent duct system at the translational level to further answer the question that whether the expression pattern of MTA1 was conservative during evolution. The western blot data were firstly measured by comparing the densitometry value of MTA1 with that of β-actin in the same experimental set. The results revealed a progressively decreasing level of MTA1 protein from caput, corpus to cauda. The specific 80 kDa band could not be observed in efferent ductules (Fig. 4A). Evaluation of the pattern of cellular localization of MTA1 protein was then carried out by using immunohistochemistry. Overall, the positive staining was relatively higher in caput and corpus when compared to that in cauda (Fig. 4B). The intense nuclear staining was predominantly found in the nuclei of principal cells (arrows). The nuclei of the basal cells (empty arrows) were also relatively weakly stained regardless of its distribution along the whole epididymis. Meanwhile, an almost negligible immunoreactivity was seen in the columnar ciliated cells (arrow heads) of efferent ductules. These results demonstrated a definite region-specific expression of MTA1 in human excurrent duct system.

### Postnatal Expression of MTA1 in the Epididymis

**Figure 5:**
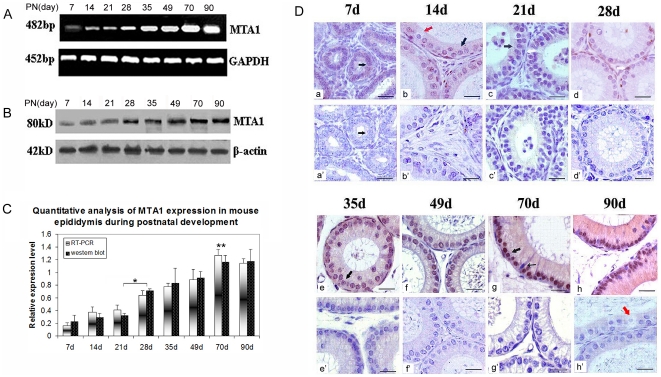
Expression of MTA1 in mouse epididymis during postnatal development. (A) RT-PCR analysis of *MTA1* expression at different time points during postnatal development. *GAPDH* was served as internal control. (B) Western blot analysis of MTA1 expression at different time points during postnatal development. β-actin was served as internal control. (C) Quantitative analysis of MTA1 expression in mouse epididymis during postnatal development by RT-PCR and western blot was carried out using band densitometry by Image J software. (* p<0.05, ** p<0.01 when comparing D70 with D21) (D) Immunohistochemistry localization of MTA1 protein in mouse proximal caput epididymis on different postnatal days 7, 14, 21, 28, 35, 49, 70, and 90 (a–h). Negative control was performed using a nonimmune serum instead of primary antibody as demonstrated in penals a'–h'. Red arrows labeled for clear cells; white arrows labeled for basal cells; black arrows labeled for principal cells, and small black arrows labeled for narrow cells. Bar = 2 µm.

Postnatal development of epididymis is a delicately organized process. Specifically, postnatal days 0 to 13 represent the proliferative phase in which undifferentiated cells undergo mitotic activity, and days 14 to 28 represent the period of differentiation when the blood-epididymal barrier is formed and the columnar cells differentiate into basal cells and principal cells, followed by a period of expansion (days 29 to 70) during which sperm enter the epididymis and are stored in the lumen of the cauda epididymis. In addition, postnatal days 20 and 40 reflect the period before and after the rise in serum androgens. Postnatal days 49 and 56 are marked by the first appearance of spermatozoa in the caput and cauda epididymis, respectively. Finally, the weight and volume of epididymis at postnatal days 90 does not increased again [17]–[18]. As a step toward understanding the developmental regulation of MTA1 expression in excurrent ducts, we followed the appearance of MTA1-rich cells in mouse epididymis during postnatal development as indicated in RT-PCR, Western blot analyses (Fig. 5A, B) and immunohistochemistry (Fig. 5D). The expression level of *MTA1*/MTA1 was relatively low before postnatal D21. There was a sharp increase of *MTA1*/MTA1 expression right after postnatal D28. This increase was latterly confirmed to be statistically significant (indicated by asterisk in Fig. 5C) using densitometry with the Image J. *MTA1*/MTA1 expression reached the highest level on postnatal D70 (indicated by double asterisk in Fig. 5C) at both transcriptional and translational level and then remained relatively steady in adulthood. Immunohistochemical staining in Fig. 5D revealed a more detailed expression pattern. Before the postnatal D21, there was a weak immunostaining of MTA1 along the entire epididymis. After postnatal D28, the epithelium began to show clear MTA1 immunoreactivity with the clear localization in basal cells (white arrow) and negligible staining in principal cells (black arrow). MTA1 staining increased gradually with a dramatic increase in the number of stained principal epithelial cells and maintained the highest level in at Day 70 (adult), concomitant with markedly elevated circulating androgen levels. Complete absence of immunoreactivity in the intertubular space and on the spermatozoa was noted in the epididymal tissue sections immunoreacted with normal goat serum or secondary antibody alone (negative control) (Fig. 5Da'-h'). Peroxidase staining increased gradually with a dramatic increase in the number of stained principal epithelial cells and clear cells, while there was a remarkable appearance of staining in the nuclei of basal cells at Day 28.

### Epididymal Expression of MTA1 Is Regulated by Androgen

**Figure 6:**
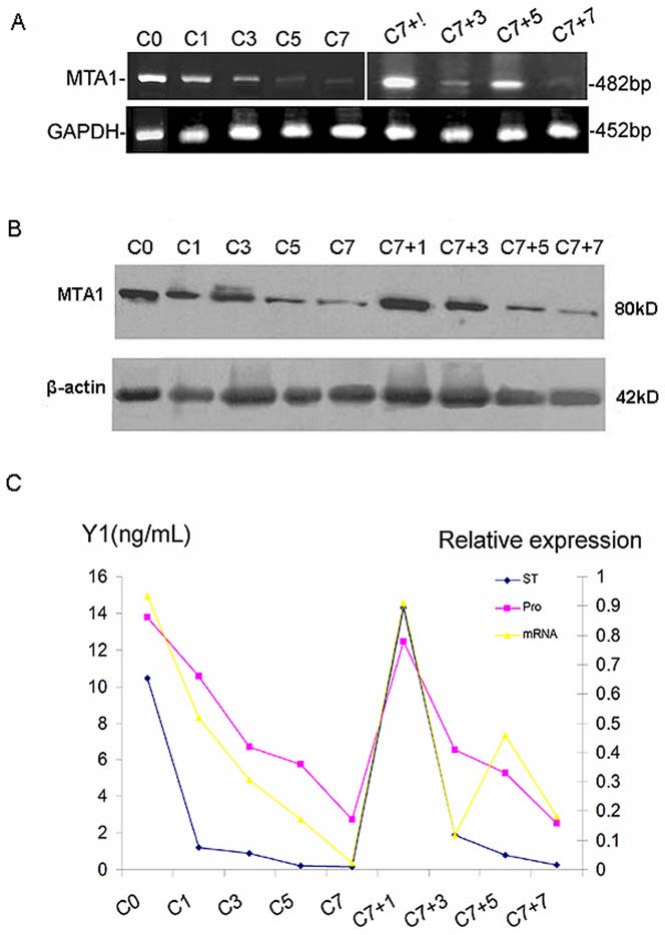
Expression level of MTA1 in mouse epididymis following castration and subsequent testosterone manipulation. (A) RT-PCR assay of adult mouse epididymis RNA from precastration (C0); bilaterally castrated for 1, 3, 5, and 7 d (C1, C3, C5, C7) and 1, 3, 5 and 7d after a single injection of testosterone propionate applied to the 7-d castrated mice (C7+1, C7+3, C7+5 and C7+7). The total RNAs were pooled from six animals at each time-point. (B) Western blot analysis of MTA1 and β-actin proteins from adult mouse epididymis at different time points as described above. (C) The relative expression levels of *MTA1* mRNA (expression density of *MTA1* mRNA/*GAPDH* mRNA) and MTA1 proten (expression density of MTA1/β-actin) in the mouse epididymis were compared with the serum testosterone level (expressed in nanomoles per liter) during androgen manipulation (n = 6).

The expression of most genes in the epididymis responds differentially to androgen [19]. Since sperm maturation is an androgen-dependent process [15], we analyzed *MTA1* gene expression under conditions of androgen manipulation. Total RNAs were obtained from the epididymides of adult mice that were sham operated, castrated, or castrated but given an injection of 5 µg/g body weight of testosterone propionate everyday for 7 days from the seventh day after castration. In the castrated animals, the serum testosterone level declined rapidly and was almost undetectable at the postoperative 7 day (Fig. 6C). In parallel, an obvious decrease was found in the *MTA1* mRNA level after surgery (Fig. 6A, C). Unexpectedly, a sharp increase of transcriptional expression of *MTA1* was detected at postoperative C7+5, which was contrasted to the relatively lower androgen level as indicated by RIA assay in Fig. 6C. This suggested that androgen might not be the solo regulator of *MTA1* expression at the transcriptional level. Western blot analysis in panel Fig. 6B confirmed the expression trend of MTA1 after castration at the protein level, with the exception of an undetected increase at postoperative C7+5 as demonstrated in RT-PCR assay. Statistical analysis revealed a clear androgen-dependent expression pattern of *MTA1*/MTA1 in the above-mentioned groups.

**Figure 7:**
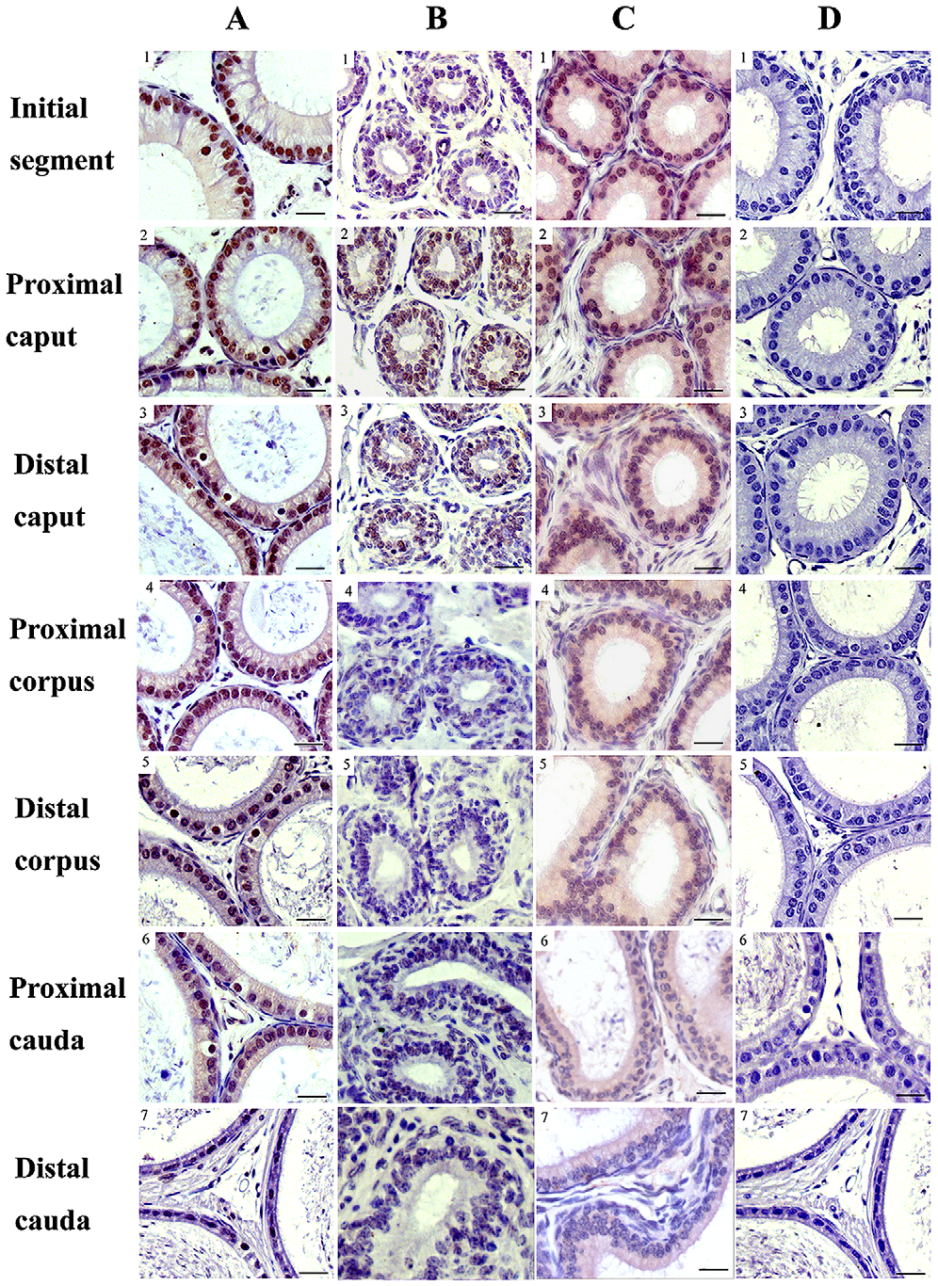
Immunohistochemical localization of MTA1 protein in the castrated mouse epididymis. Sections of the epididymal segments from various groups of mice including sham group (A) were immunolocalized with a MTA1 antibody and nonimmune goat serum (negative control) (D) as described under “Materials and Methods” and counterstained with hematoxylin. The data depicted represents staining for the MTA1 protein in epididymis of day 7 postcastration mice (B) with testosterone propionate supplementation (day 7) (C). Bar = 20 µm.

Subsequent histological analysis gave us a more detailed expression pattern of MTA1 after surgery (Fig. 7). Overall, in the castrated animals, a significant decrease in the luminal diameter of the epididymis was observed at a high magnification (Fig. 7B), consistent with previous RIA result. This morphological change was more pronounced in the initial segment, caput, corpus followed by cauda epididymis. The expression of MTA1 protein decreased significantly in the surgical group (Fig. 7B) when compared to that of sham operated group (Fig. 7A). However, the expression of the protein could be restored following supplementation with androgen (Fig. 7C). Substitution of primary antibody with non-immune serum completely abolished the positive staining (Fig. 7D), confirming the specificity of this assay.

## Discussion

The results from the present study showed that there were region-specific changes in the mRNA and protein expression of MTA1, primarily in the epithelia, but not in the luminal contents of the epididymis. The highest levels of MTA1 expression were found primarily in the principal epithelium of the caput and the proximal corpus, whereas the levels were markedly reduced in the distal corpus and the cauda of the epididymis, indicating a possible involvement of MTA1 during early and late sperm maturation instead of contributing to the storage of mature spermatozoa [20]–[22]. Additionally, similarity of its appearance in adult human epididymis, also lends support to our speculation. Interestingly, mainly nuclear staining and regional cytoplasmic staining (caput and corpus) were detected by anti-MTA1 antibody (Fig. 2B). Since MTA1 belongs to nucleosome remodeling and deacetylation (NuRD) complexes and could repress the transcription of different genes by recruiting histone deacetylases onto their target genes and it also acts as a coactivator in a promoter-context dependent manner [23], it is not surprising that immunohistochemical assays have documented the predominantly nuclear localization of MTA1 in various segments of mouse epididymis.

Within the mature epididymis, the principal cells outnumber all the other cell types combined by at least 3∶1 [24]–[25]. Principal cells appear generally very active with respect to transport and secretion of small organic molecules, absorption of fluid, and protein synthesis and secretion [26]. Clear cells contain the vacuolar H+-ATPase and secrete protons into the lumen and thus participate in its acidification. In addition, they are also endocytic cells and may be responsible for clearance of proteins from the epididymal lumen [27]. MTA1 was mainly immunolocalized in the nuclei of principal and clear cells, both of which show endocytic role in normal adult animals, as demonstrated by immunofluorescence assay [28]. Actually, yeast two-hybrid system has identified MTA1 as an interacting partner of endophilin 3 which participates in the process of endocytosis [29]. On the above basis, we speculate that MTA1 possibly participated in the transcriptional regulation of secretory and endocytic genes within the epithelial cells.

Androgen is essential for maintenance of spermatogenesis in the testis and for maturation of spermatozoa in the epididymis which will be reduced to 25% of its normal weight following castration [30]–[31]. In the present study, serum testosterone declined rapidly and was almost undetectable from the first day. A clear decrease was correspondingly found in the MTA1 mRNA and protein level on the third day post-castration and it was nearly undetectable by day 7 after surgery. Since bilateral orchidectomy lowers blood testosterone concentrations to undetectable levels, the dramatic decrease in MTA1 levels following castration convincingly suggested that testosterone may play a regulatory role in the modulation of MTA1 expression. Testosterone replacement for the animals 7 days after castration resulted in a rapid increase in the serum testosterone concentration and the MTA1 expression level in the epididymis. However, a sharp increase of transcriptional expression of *MTA1* was unexpectedly detected at the 5th day of testosterone treatment. These observations collectively indicated that *MTA1* expression was actually modulated by androgen signaling but this expression change may also require a posttranscriptional modification under certain pathological conditions. Actually, it has been reported that *MTA1* is a DNA-damage response gene and regulates p53-dependent DNA repair [14]. P53 is important in the regulation of cell production during normal spermatogenesis either by regulation of cell proliferation or more likely, by regulating the apoptotic process [32]. This may account for the unexpected increase of the *MTA1* expression inconsistent with the low level of circulated androgen at the 5th day after testosterone supplementary treatment since castration is an intrinsic stress condition. Since MTA1 is required for the p53-dependent DNA repair and p53 is believed to be a guardian of genome integrity during normal spermatogenesis [13]–[14], findings presented here in conjunction with the earlier finding of a crucial role of MTA1 in DSB repair suggest a potential role of MTA1-p53 in epididymal function. This androgen up-regulated expression pattern also provides a logical explanation for why levels of MTA1 mRNA in the young mouse testis were much lower than those in adults [12]. The contribution of such a phenomenon to the documented effects of androgen regulation remains to be elucidated. Nonetheless, being a key member of the nucleosome-remodelling and –deacetylation (NuRD) complex, the androgen regulation of the MTA1 protein indicates that the *MTA1* gene might be involved in a cascade of androgen-regulated events in the epididymis and especially attains considerable significance in the realm of epididymal sperm maturation.

The gradual and significant increase in *MTA1/*MTA1 expression was observed during postnatal development of the mouse epididymis. Similar expression pattern was also observed in other epididymal proteins such as acidic epididymal glycoprotein [33] and protein SP [34]. During the course of maturation in the mouse, the production of testicular testosterone (T) and possibly its availability to the target organs increase abruptly from 20 to 40 days of age [35]. Nevertheless, the expression level of *MTA1/*MTA1 was relatively low before postnatal D21 and increased dramatically from postnatal D28 (Fig. 5). This was in tandem with the acquisition of hormonal maturation of the epididymis with increases in age. By day 35, the principal cells of the epididymis attain adult-like structural features along with high levels of luminal androgens [36]. The appearance of MTA1 protein in the principal cells from day 35 coincides with the complete differentiation of principal cells from columnar cells and their further differentiation to principal cells and apical cells. The fact that the MTA1 protein was barely detectable in the nuclei of principal cells before postnatal days 35, despite high concentrations of androgens and AR mRNA [35], indicated that the differentiating principal cells may simply be unable to express *MTA1* mRNA. Very interestingly, we found MTA1 protein was clearly localized in the basal cells at postnatal day 28, coinciding with the initial differentiation of basal cells from columnar cells [37]. Basal cells do not access the luminal compartment and are in close association with the overlying principal cells, and have the remarkable property of extending long and slender cytoplasmic projections that cross the tight junction barrier to monitor the luminal environment [38]. The contribution of basal cells in differential period to the testicular expression of MTA1 is yet to be determined. However, the above-mentioned features of the mouse MTA1 strongly indicate that it has unique functions in the epididymis, perhaps associated with the differentiation of different cell types at specific developmental stage.

In conclusion, the outcome of our study suggests that *MTA1/*MTA1 expression is present in the entire mouse epididymis, especially in the proximal segment, and its expression is regulated by androgen and the developmental status of the epididymis. Future studies deserve to be directed to further delineate the physiological relevance of this histone modifier in androgen-regulated epididymal function.

## Materials and Methods

### Ethics Statement

The Ethics Committee for Animal Experiments of the Fourth Military Medical University approved all animal work (Permit number: 10001) and the experimental protocols strictly complied with the institutional guidelines and the criteria outlined in the “Guide for Care and Use of Laboratory Animals”. All surgery was performed under sodium pentobarbital anesthesia, and all efforts were made to minimize suffering. For human samples, the informed written consent have been obtained from the immediate families of three victims of car accidents (please see details below). The use of the human tissue in this study was approved by the Human Research Committee of the Fourth Military Medical University for Approval of Research Involving Human Subjects.

### Animals and Tissue Preparation

Adult male mice (BALB/c) were obtained from the Animal Research Centre of the Fourth Military Medical University, Xi'an (China) and maintained on a 12-hour light: 12-hour dark in a 20–25°C environment. They were allowed to acclimatize for at least 1 week before the experiment. Tissues including testis and epididymis were collected immediately after the animals were sacrificed. The latter was dissected under stereo microscope (Omano OM4413) and divided into three regions: the caput (including efferent ductules), corpus, and cauda [39]. For SDS-PAGE and western blotting, the tissue lysates were prepared as previously described [10]. Briefly, tissues were homogenized using RW16 Basic S1 Overhead stirrer (IKA® works Inc., Wilmington NC) in Laemmli buffer, containing Tris-Glycerol with 5% β-mercaptoethanol, 2% SDS and 100 mM PMSF (Phenyl methyl sulfonyl fluride). Subsequently, the lysate was centrifuged at 14000 rpm for 15 min at room temperature and the supernatant containing the total lysate protein was stored at −80C° until use. For histological studies, mice were perfused with Bouin's fixation through left ventricle. The tissues were post-fixed in fresh Bouin's–PBS (pH 7.4) for another 2 h, dehydrated through graded ethanols, embedded in paraffin and further processed into 4-µm-thick sections for the following immunohistochemical examination.

### Human Tissue Samples

Normal human testes and epididymis were obtained from three victims of traffic accidents aged 25–40 years as described in previous publication [40]. To ensure the usability of the tissues, normal spermatogenesis was confirmed in all samples in accordance with the criteria by Suarez-Quian et al [41].

### Castration and Testosterone Supplementation

90 ten-week-old normal male BALB/c mice were castrated bilaterally. The sham operated mice were served as the control group. Animals were divided into nine groups (six mice per group) and killed on Day 0, 1, 3, 5, and 7 after castration as well as 1, 3, 5, and 7 days after the initial testosterone propionate injection. Androgen supplementation began on the seventh day after castration with a single injection of testosterone propionate (5 µg/g bodyweight). Pooled serum samples from each group were measured for testosterone concentration by RIA as described previously [42].

### Isolation of Mature Spermatozoa and Luminal Fluid from Mouse Cauda

Sperm cells were collected following the reported method [43]. Briefly, cauda epididymidis was excised and then rinsed with medium containing 150 mM NaCl, 5 mM KCl, 2 mM CaCl_2_, 1 mM MgCl_2_, 30 mM HEPES, 10 mM glucose, 10 mM lactic acid, and 1 mM pyruvic acid (pH 7.4). After transfer to 1 ml of medium supplemented with 5 mg of bovine serum albumin per ml and 15 mM NaHCO_3_, semen was allowed to exude (15 min at 37°C, 5% CO_2_) from three to five small incisions. Cells were diluted to 4 ml and collected twice by sedimentation (400× g; 5 min). To obtain the luminal fluid, the cauda epididymidis was excised and placed in 0.05 M Tris buffer, pH 6.8, in a watch glass. Micropuncture was subsequently performed to allow the fluid gently expressed into the buffer. The spermatozoa were removed by centrifuge for 5 minutes at 6000× g as described elsewhere [44].

### Developmental Study

To determine if MTA1 could be involved in developmental as well as mature functions in mouse epididymis, expression of the MTA1 mRNA and protein was analyzed in the tissues from animals of different ages. The animals (number) were sacrificed on postnatal days 7 (8), 14 (8), 28 (6), 35 (6), 49 (6), 56 (6), 70 (6) and 90(6) days. Epididymal tissues obtained from each postnatal age were pooled into three groups in order to obtain samples in triplicate. The time points chosen here were coinciding with known developmental events [12].

### RNA Isolation

Total RNA was isolated from testis, three regions of the epididymis and isolated spermatozoa using TRIzol Reagent (GIBCO BRL, Gaithersburg, Md., USA) [10]. All steps during isolation were performed according to manufacturer's instructions. The quantity of RNA was estimated spectrophotometrically at OD260. Additionally, the integrity of the isolated RNA was checked on a 1% (w/v) MOPS–formaldehyde agarose gel.

### Semi-Quantitative RT-PCR

Two microgram of total RNA was reverse transcribed (RT) to obtain complementary DNA (cDNA) using reverse transcriptase and random hexamers (Invitrogen) according to the manufacturer's instructions. Polymerase chain reaction (PCR) analysis was performed using the forward primer (5′-GGA GTG GTC CGC ATC AGA-3′), and the reverse primer (5′-CTA ACC GGG TTG GCA TTT-3′) for *MTA1* (GenBank: AF288137). Expression value from the housekeeping gene glyceraldehyde-3-phosphate dehydrogenase (*GAPDH*; GenBank: DQ403054) gene was used for normalization. The sequences of primers for GAPDH were: 5′ GCC TCA AGA TCA GCA AT 3′ (forward) and 5′ AGG TCC ACC ACT GAC ACG TT 3′(reverse). The mouse protamine 2 (*prm-2*; GenBank: NM_008933.1) was served as positive control for identification of the purified caudal spermatozoa. The sequences of primers for prm-2 were: 5′ TCA TCA CCA CCA AGA GCA GGT GG 3′ (forward) and 5′ GCT TTA TTT GGC AGG TGG CTT TGC T 3′(reverse). The PCR protocol used were as follows: initial denaturation at 94°C for 5 min, 32 cycles of denaturation at 94°C for 30 sec, annealing at 58°C for 40 sec and extension at 72°C for 30 sec and a final extension at 72°C for 10 min in the total volume of 25 µl. The PCR amplification products were resolved on 1.5% agarose gel and visualized under UV light after 0.5% ethidium bromide staining. The anticipated sizes of the amplified fragments were 482 base pairs (bp) for *MTA1*, 452 bp for *GAPDH* and 601 bp for *prm-2*. All samples were run in triplicate at least to illustrate the typical result. In all assays, liquid controls and reactions without RT resulted in negative amplification. Glyceraldehyde-3-phosphate dehydrogenase (*GAPDH*) was used as an internal control for each of the PCR amplifications.

### Western Blot Analysis

After tissue preparation, the protein concentration was determined with the BCA (bicinchoninic acid) assay (Pierce, Rockford, III) according to the manufacturer's instructions. Protein samples were diluted in loading buffer, boiled for 5 min, loaded onto gels at 40 mg per lane. The proteins were separated in 10% SDS-PAGE, followed by transfer to NC membrane which was subsequently blocked with 5% nonfat milk and 0.1% Tween-20 in Tris-buffered saline, pH 7.4, for 1 h at room temperature, and incubated with the MTA1 antibody (dilution 1/400 in blocking solution; Santa Cruz Biotechnology, Santa Cruz, CA, USA) overnight at 4°C. The membrane was then rinsed with PBS and incubated with horseradish peroxidase conjugated donkey anti-goat IgG (dilution 1/4,000 in blocking solution) for 45 min at room temperature, followed by three rinses with PBS. The horseradish peroxidase was visualized by using a chemiluminescence substrate (ECL plus Western blot detection system, Pierce Biosciences). The blots were subsequently stripped and reprobed with a β-actin monoclonal antibody as an internal control to confirm equal loading of protein samples in the gel.

### Immunohistochemical and Confocal Immunofluorescence Microscopy Study

Streptavidin-biotin complex (SABC) immunohistochemical method was conducted as previously described [11]–[12]. In brief, the sections were exposed to 0.5% hydrogen peroxide in methanol for 30 min to destroy endogenous peroxides activity after deparaffinization and rehydration. The slides were then incubated with the anti-MTA1 goat antibody (Santa Cruz Biotechnology, Santa Cruz, Calif., USA; 1∶50 dilution) diluted in PBS, at 4°C overnight in a moist box. Biotinylated rabbit anti-goat IgG (1∶200 dilution; Sigma) was incubated on the sections for 1 h at room temperature and detected with streptavidinperoxidase complex. Peroxidases were detected with 0.7 mg/ml 3-3′-diaminobenzidine tetrahydrochloride (Sigma, St. Louis, MO, USA) in 1.6 mg/ml urea hydrogen peroxide. The sections were then counterstained with hematoxylin for 60 sec. Control slides were incubated with a nonimmune serum instead of primary antibody.

For immunofluorescence staining, the method was as described elsewhere [45]. The primary antibodies were goat anti-mouse MTA1 antibody (dilution 1∶100), chicken anti-ATP6E immunoglobulin Y (IgY) antibody (dilution 1∶200; Genway Biotech) labeled for clear cells, and rabbit anti-CLU antibody (dilution 1∶100; Santa Cruz) labeled for principal cells [15]. The second antibodies were fluorescein isothiocyanate (FITC)-labeled anti-goat IgG (dilution 1∶300;Sigma), Rhodamine-conjugated bovine anti-chicken IgY (dilution 1∶200; Santa Cruz) and Rhodamine-conjugated goat anti-rabbit IgG (HtL) (dilution 1∶200; Jackson Immune Research Laboratories), and Rhodamine-conjugated anti-goat IgG. The sections were mounted in 80% glycerol and examined with a confocal microscope (Leica TCS SP2 AOBS).

### Statistical Analysis

Experiments were repeated at least three times, and one representative from at least three similar results is presented. The significance of the results was determined by using the one-way ANOVA parametric test. Statistical differences were considered significant at P<0.05. Data were presented as the mean ± SD.
